# Thank You—A Thousand Times

**DOI:** 10.1523/ENEURO.0174-19.2019

**Published:** 2019-05-22

**Authors:** Christophe Bernard

**Affiliations:** Editor-in-Chief

This is a special time for the journal. We published our 1000th paper. This success belongs to our community of neuroscientists. Since launching *eNeuro*, just before the Society of Neuroscience annual meeting in 2014, we have come a long way.

It is always difficult to launch a new journal as its success depends upon the trust scientists have in the journal. Of course, *eNeuro* is a society journal with society values: quality of science, consideration for authors first, run by active research scientists, transparency, fairness, and no financial interest. But holding such values does not guarantee that the journal will be chosen by fellow scientists. *eNeuro* was.

The number of submissions is still on the rise. The number of accesses to published articles is increasing exponentially, demonstrating that the science published in *eNeuro* has a large impact in the community. Feedback from authors and reviewers tells us they are very happy with the way we are handling manuscripts and how we reach a consensus decision in review to present to authors.

Over the years, we have introduced many innovations, placing *eNeuro* among the forefront of journals seeking to change how science is published. In the coming months, more innovations will be announced. My goal remains the same as it was at launch, that is to make *eNeuro* the flagship of online-only, open access neuroscience journals. We are getting there. So, to all, thank you. A thousand times.

